# Adhesive Joints with Laser Shaped Surface Microstructures

**DOI:** 10.3390/ma14247548

**Published:** 2021-12-09

**Authors:** Szymon Tofil, Robert Barbucha, Marek Kocik, Rafał Kozera, Mateusz Tański, Natarajan Arivazhagan, Jianhua Yao, Andrej Zrak

**Affiliations:** 1 Laser Research Centre, Faculty of Mechatronics and Mechanical Engineering, Kielce University of Technology, Av. Tysiąclecia P.P. 7, 25-314 Kielce, Poland; 2 Institute of Fluid Flow Machinery, Polish Academy of Sciences, Generała Stanisława Fiszera 14, 80-231 Gdańsk, Poland; brobert@imp.gda.pl (R.B.); kocik@imp.gda.pl (M.K.); tanski@imp.gda.pl (M.T.); 3 Faculty of Materials Science and Engineering, Warsaw University of Technology, Woloska 141, 02-507 Warszawa, Poland; rafal.kozera@pw.edu.pl; 4 School of Mechanical Engineering, VIT University, Vellore 632014, India; narivazhagan@vit.ac.in; 5 Institute of Laser Advanced Manufacturing, Zhejiang University of Technology, Hangzhou 310023, China; laser@zjut.edu.cn; 6 Faculty of Mechanical Engineering, Žilinská Univerzita v Žiline, Univerzitná 8215/1, 010 26 Žilina, Slovakia; andrejzrak@gmail.com

**Keywords:** picosecond laser devices, surface micromachining, plastics, adhesive joints, microstructure, laser cold ablation, modification of the surface layer, laser micromachining, UV laser

## Abstract

One of the most commonly applied methods of joining dissimilar materials is gluing. This could be mainly attributed to the applicability of this technique in various industries. The article presents a method of material surface treatment, which increases the shear strength of adhesive joints for lightweight metals such as aluminum with plastics. For this purpose, laser surface microstructuring was performed on each of the selected construction materials. As a result of the performed treatment, the active surface of the glued area was increased, which increased the adhesive strength. The picosecond laser with UV radiation used in the research is TruMicro 5325c with which material can be removed as a result of the cold ablation phenomenon. The applied parameters of the laser device did not cause thermal damage to the surface of the microstructured materials, which was confirmed by microscopic examination. Laser micromachining did not deteriorate the degree of wetting of the tested materials, either, as was confirmed by the contact angle and surface energy measurements with the use of water as the measuring liquid. In investigated cases of microstructure types, the presented method significantly increased the shear strength of the joints formed, as demonstrated by the presented strength test results. Research has shown that created joints with microstructure made according to the described method, are characterized by a significant increase in strength, up to 376%, compared to materials without microstructure. The presented results are part of a series of tests aimed at selecting the operating laser parameters for the implementation of geometric shapes of microstructures which will increase the strength of adhesive joints in selected materials.

## 1. Introduction

One of the most commonly applied methods of joining dissimilar materials is gluing [1,2,3,4]. For over 80 years, adhesive joints have been extensively researched because they are widely used as one of the methods of joining identical and dissimilar materials [5]. It is always important to develop a joint with maximum safety enhancement adding minimal weight to the structure for low energy consumption with concern for the environment. However, methods of joining two different materials, such as fasteners including bolts and rivets, and adhesive bonding, such as welding and soldering, have always caused serious problems that degrade the materials. While advantages of fasteners are simple processing, high joining strength, and small scatter in the data, disadvantages include increase in weight due to the fasteners and low sealing performance. Moreover, the bolt holes decrease the cross-sectional area and can act as stress concentrators. It has been reported that drilling holes in Fiberglass Reinforced Plastic (FRP) laminate composites results in breakage of the reinforcing fibers, peeling of the top plies at hole entry, resin degradation at the hole wall, and delamination of the bottom plies of the laminates [6,7]. The resulting damage can result in generation of fatigue cracks during fatigue [8]. The technology of joining homogeneous and dissimilar surfaces with the use of the adhesion phenomenon is currently widely applied, especially in industrial sectors requiring precision, strength and lightness of joints. It is primarily the aviation industry where the need for the technology of adhesive joints and gluing was created by the introduction of new, lightweight construction materials [9,10,11,12]. The laser treatment of polymeric materials has been described in many scientific publications [13,14,15,16,17]. Over the last decade, industry has used laser devices even more frequently [18,19], as is widely described in scientific papers from various fields [20,21,22,23,24]. Laser devices significantly facilitate the production work, among others, in the aviation, automotive and electronic industries [24,25]. Rapid development of lasers has made it possible to perform various modifications, not only in the surface layer of typical materials [22,23,24], but also those that until recently were considered difficult to machine. Currently, widely understood laser micromachining is more and more commonly used to modify surface properties in various construction materials, which has been described in the following research works [26,27,28,29,30].

The most commonly used materials are polymers, composites and aluminum alloys, the use of which ensures lightweight construction while maintaining high strength and stiffness, and at the same time low material density. Aluminum (Al) with its light specific weight and high corrosion resistance due to passivation is a valuable lightweight structural material utilized for aerospace technology with high specific strength. Polycarbonate (PC) on the other hand, is an engineering plastic of high transparency having mechanical strength more than 150 times that of tempered glass. Because PC is lightweight and exhibits superior properties such as workability, impact resistance, and withstands harsh weather conditions as well, it is widely used for protective articles including cockpit windows, building structures and covers for electronic equipment. The use of plastic materials allows for great freedom of detail geometry during the design and treatment [31,32] and gluing is often the only possible method of joining them. Especially in aviation and automotive industry, the continuous improvement of the current and the occurrence of new construction solutions necessitate application of novel developments in the field of materials engineering. The use of laser techniques seems to be both justified and very promising [33,34,35]. The direct impact of the laser beam on the surface of various materials has been the subject of many scientific studies presented in various publications [36,37,38,39]. These works investigated how even the slightest changes in laser operating parameters (such as: wavelength, frequency, pulse duration, etc.) affect the surface of the processed material. The use of various types of laser devices and thus various techniques for modifying the surface of light metal alloys and polymeric materials are presented in more detail in [40,41,42].

Along with the rapid development of technology, the offered products must meet much higher requirements than before. This mainly concerns changes in durability [39], production costs, energy and material consumption. Methods used presently by a majority of manufacturers involve welding and mechanical joining. Unfortunately, these methods are not always the most practical solutions for modern assembly. In particular, mechanically connected elements often increase production costs, the weight of structure, and impose restrictions on the possible materials choice. In addition, they lead to premature fatigue failure of the material, deformation or cracks. The use of strong adhesive can outperform mechanical joints in many structural applications. Therefore, a growing group of manufacturers from various industries are looking for new types of industrial adhesives as a replacement for traditional joining methods. Achieving a high level of reliability of various technical objects is often possible thanks to the use of parts of products used as nondismountable units, in which the adhesion technique is widely used.

The aim of this work is to present the effect of the type of laser-produced microtexture on the surface of the material on the increase of the adhesive strength in glued joints. The conducted preliminary tests showed that inadequate preparation of the surface before gluing significantly deteriorates the strength of the joint. Especially in the case of the laser modified area. Too much heating of the material (especially metal) causes the accumulation of oxides on its surface, which weaken the adhesion of the glue. The use of the fs laser does not cause excess energy, which is converted into heat causing thermal changes in the material. Performing the treatment without leaving any contaminants on the surface of the material is crucial for the correct gluing process. The article presents the possibilities and effects of using the laser technique to create microstructures on the surface of the tested material and thus obtain a more durable joint. AW7075-T6 aluminum alloy and polycarbonate were used in the tests, and joined with the Multibond 1101 epoxy adhesive.

## 2. Materials and Methods

**Figure 1:**
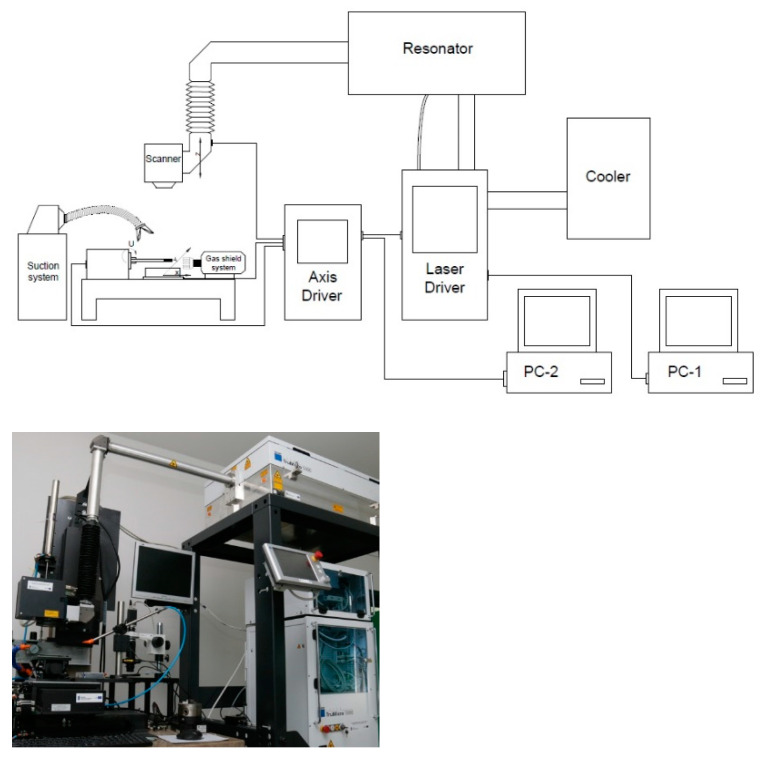
Diagram and view of the laboratory stand with the TRUMPF 5325c laser used in the research.

The surface microtexturing of the tested materials was carried out on a laboratory stand located in the Laser Processing Research Center at the, Kielce University of Technology (Kielce, Poland). The station is equipped with a TRUMPF 5325c laser that generates picosecond pulses of the UV beam with a wavelength of 343 nm. The laser beam on the surface of the material was guided using the SCANLAB intelliSCAN 14 scanning head. The general view of the laboratory stand is shown in Figure 1.

**Figure 2:**
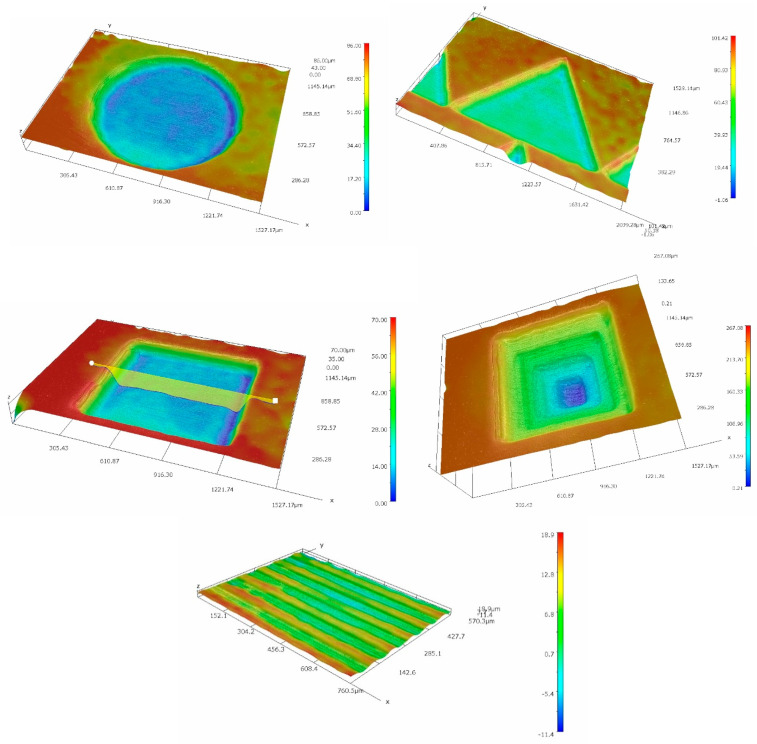
General view of the shape of the single microstructures.

**Figure 3:**
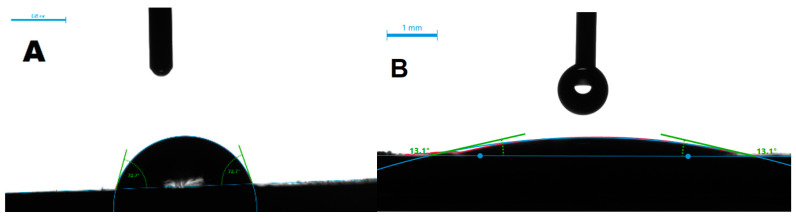
An example of the measurement view of the contact angle of the surface for a sample made of aluminum—(**A**) without texture and (**B**) with E variant texture.

**Figure 4:**
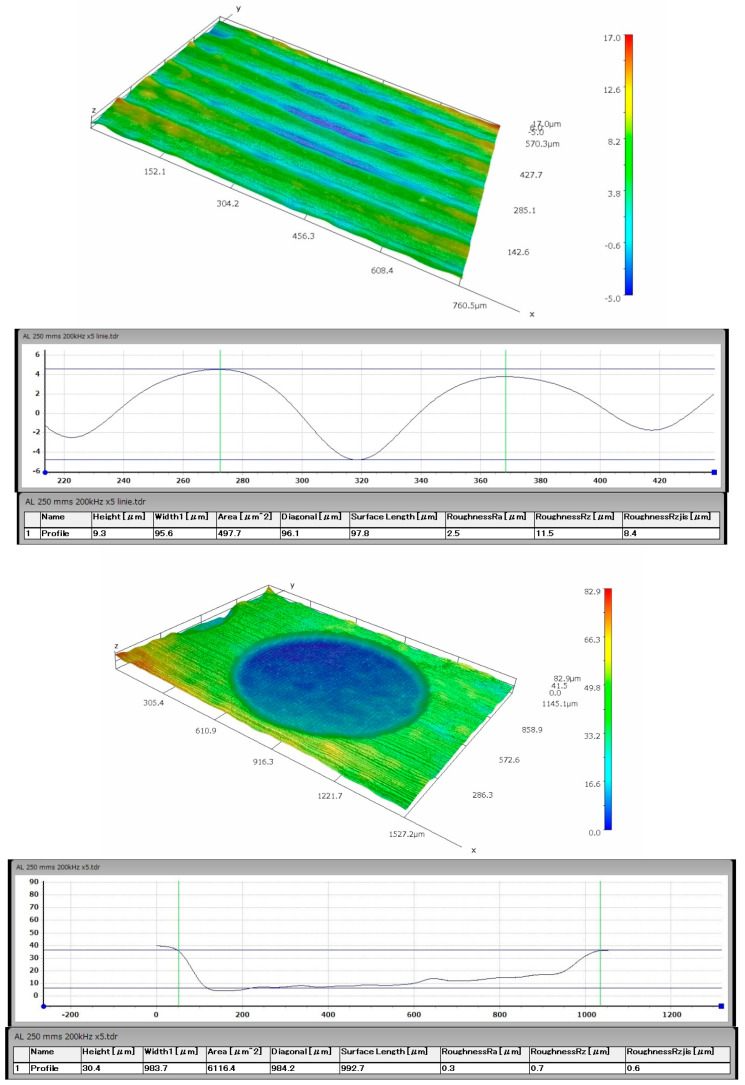
An example of the aluminum surface after type A and E micromachining.

**Figure 5:**
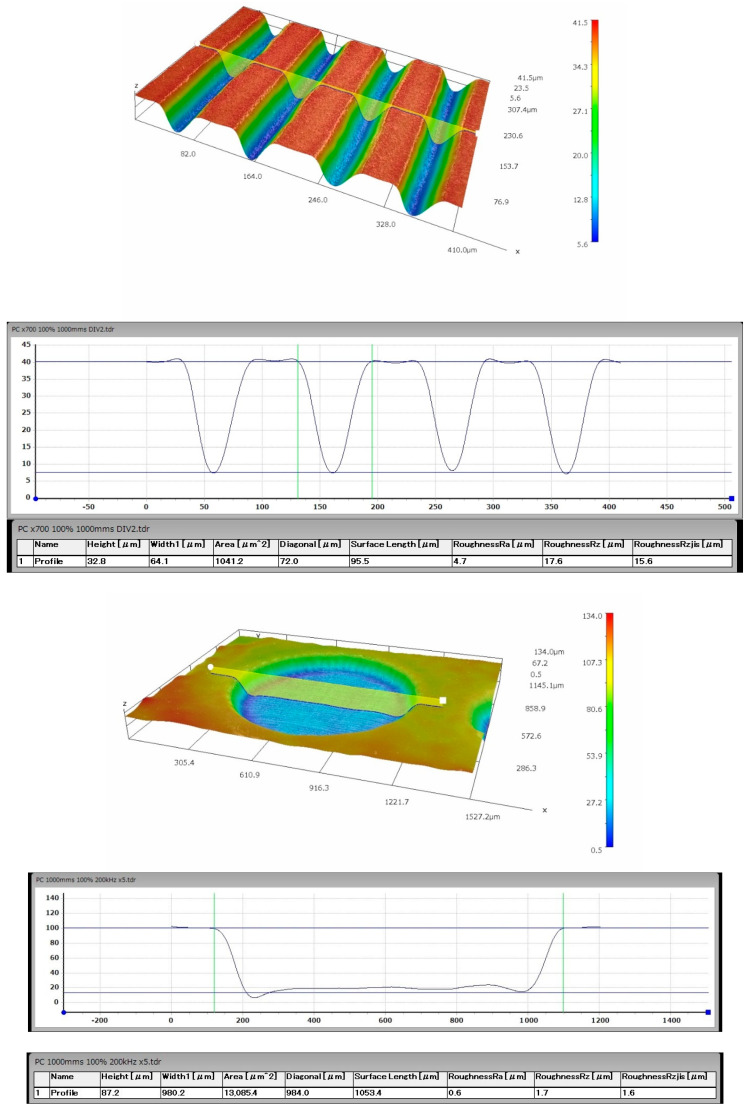
An example of the PC surface after the type A and E micromachining with the surface profile and geometric measurements.

**Figure 6:**
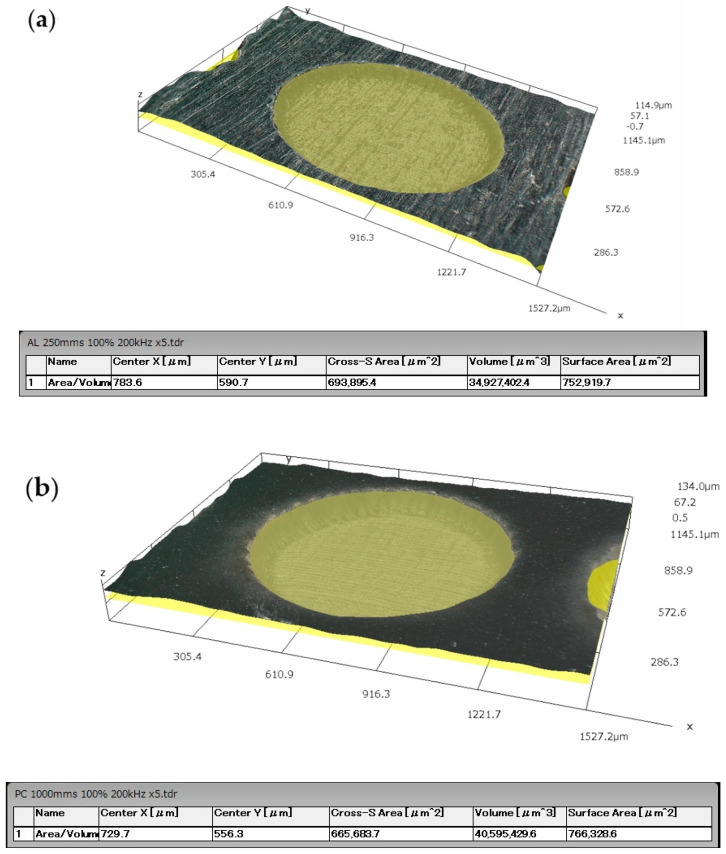
A screenshot example of performing a volume measurement after the type A and E micromachining on the Al (**a**) and PC surfaces (**b**).

**Figure 7:**
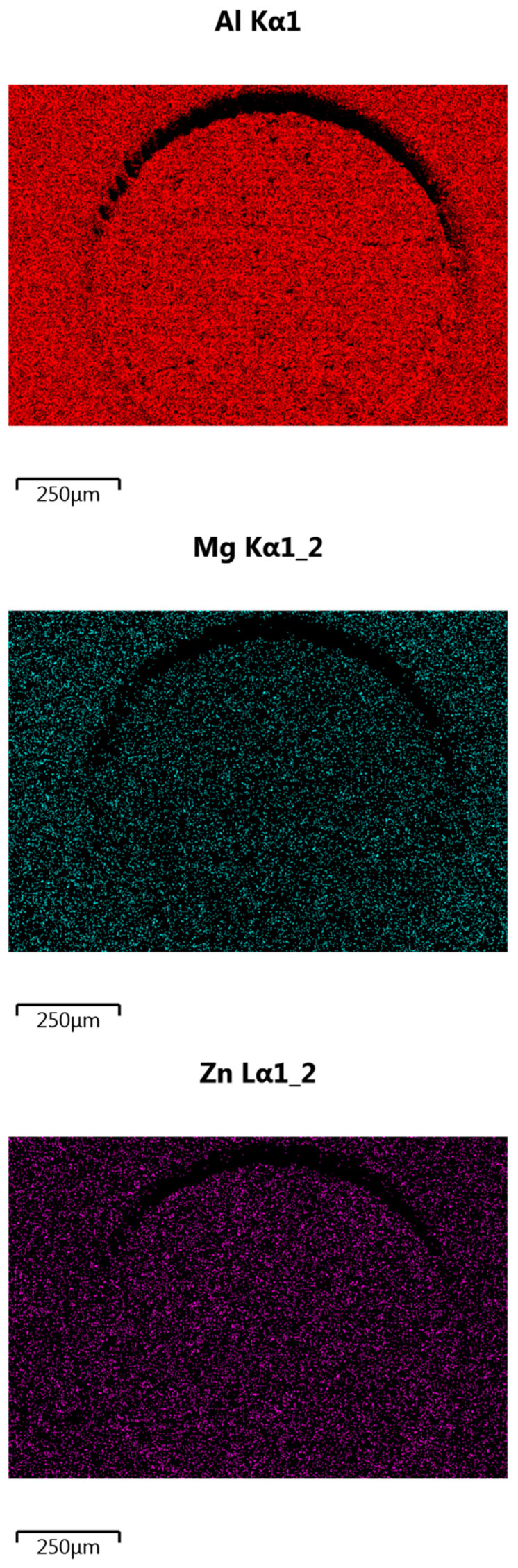

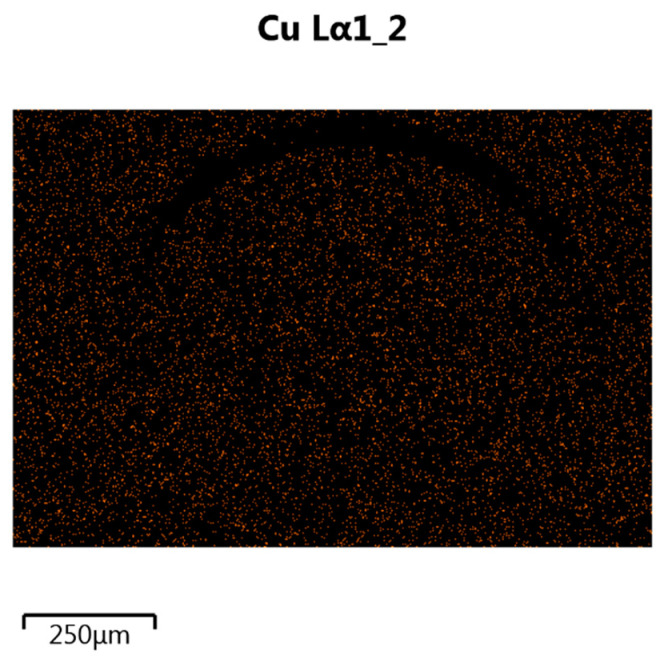
An example of the analysis result of the distribution of selected elements on the surface of the tested sample.

Five variants of microtexture were made, the shapes of which are shown in Figure 2. For each geometric shape, the microtexture was preceded by the study of the surface free energy and the contact angle of the surface [43,44,45,46], as shown in Figure 3. The tests were performed with the use of the Goniometer DataPhysics OCA 15 in accordance with the PN-EN 828: 2013-05 standard. The obtained effects of the micromachining performed for selected microtextures are shown in Figure 4, Figure 5, Figure 6 and Figure 7. For surface diagnostics purpose, the HIROX KH-8700 optical microscope (Hirox Co., Ltd., Tokyo, Japan) and the JOEL 7100f scanning electron microscope (JEOL Ltd., Tokyo, Japan) were used. 

**Figure 8:**
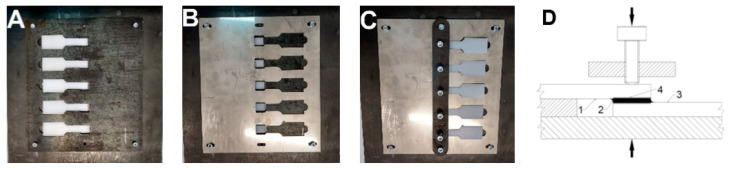
View of the subsequent stages of gluing the samples placed in a holder specially designed for this purpose, and the connection diagram.

**Figure 9:**
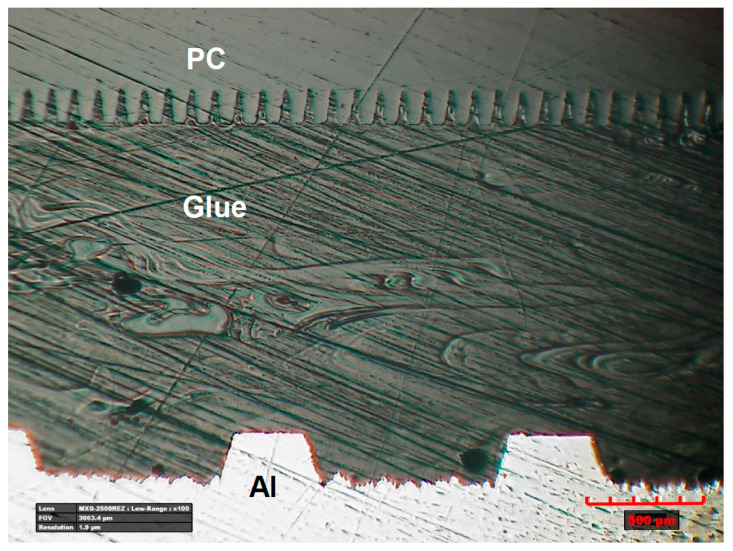
General view of the joint cross section with additional adhesive for the Al-PC.

**Figure 10:**
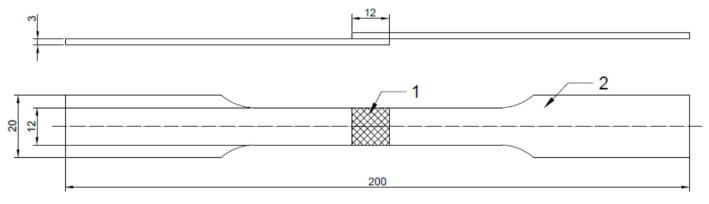
Diagram of the elements prepared for strength tests joined overlapping. Above—side view, and below—top view, unit: mm.

The tested materials were joined with the Multibond 1101 epoxy glue. The joining process is shown in Figure 8, and the transverse view of the joined elements is shown in Figure 9. The endurance tests were carried out using the INSTRON 4502 machine (Norwood, MA, USA). The diagram of the elements prepared for the overlapping strength tests is presented and described below (Figure 10). The performed tests allowed to determine how the micromachining of the selected geometric shape increased the force needed to break the connection.

The experimental setup consisted of a TruMicro 5325c laser source with the ScanLAB GALVO intelliSCAN 14 head and a worktable where the laser beam is delivered. The scanner head was equipped with an F-Theta lens with a focal length of f = 160 mm. The worktable consists of automated linear X, Y, Z and rotary A axes by Aerotech. The accuracy of the X, Y, Z axes is 0.0001 mm and the A axis accuracy is 0.1°. Operating parameters of the laser device used during surface treatment of the tested materials: average power: 5 W; pulse energy: 12.6 µJ; pulse frequency: 200–400 kHz; pulse duration: 6.2 ps; beam scanning speed: 250–2000 mm/s. 

In Figure 2, a general view of the shape of the single microstructures is presented. Microtextures in the shape of a truncated cone, a cuboid with a square base and an equilateral triangle, an inverted pyramid (a pyramid with a square base) and parallel lines with an isosceles triangle cross-section were made.

The micromachining effect was obtained by material ablation from the sample surface using the linear beam trajectory without overlap of the beam in each steps. This procedure was used to make all five microtexture variants. It is one of the possibilities of microtexture provided by the setup used. Further studies have been scheduled to take into account different variants of the compaction and depth of the laser-made microtexture on other polymer materials. The results will be published in the subsequent papers of the research team. 

Table 1 shows the operating parameters of the TruMicro 5325c laser device used to make microtextures. Table 2 presents the geometrical characteristics of individual surface structures.

For the various geometrical variants of the microtexture (Figure 2) presented above, measurements of the surface free energy (SEP) and the contact angle were performed. The liquid used to perform the wettability tests was water. The tests were performed by placing drops of 2 µL volume with a computer-controlled dispenser on the substrates of the tested laser-modified materials. Measurements were made for drops placed in a single microtexture area. The drops were deposited using the so-called the droplet released method, in which it is released from the applicator as soon as it comes into contact with the substrate. The contact angle between the sample surface and the tangent plane to the drop surface was measured after the equilibrium conditions had been established. The equilibrium state was assumed to be the conditions in which the value of the contact angle stabilized (i.e., it stopped decreasing as a result of the droplet spread over the surface). Using the built-in analysis software, the value of the contact angle formed by the droplet with the test substrate was read, and the surface free energy value was measured for each pattern. The test was performed at room temperature based on the equation of state. Table 3 presents the obtained measurement results immediately after the laser micromachining.

For example, for aluminum samples, the parallel-lines-shaped microtexture with an isosceles triangle cross-section (type E) changed the surface properties to super hydrophilic, as can be seen in Figure 3. For the truncated cone-shaped microtexture (type A), the selected materials did not show hydrophobic properties. Other presented variants of the shape of the microtexture (type B, C, D) were eliminated at the stage of wettability tests, due to the insufficient change in the degree of surface wetting in relation to the reference sample. For the gluing process and for strength tests, wet-texture in the shape of a truncated cone (type A) and parallel lines with an isosceles triangle cross-section (type E) were selected.

## 3. Results

### 3.1. Micromachining Results

According to the above-described procedure, E-type microstructures perpendicular to the breaking force with a spacing of about 50 µm and an average depth of 25 µm for PC and 15 µm for aluminum were made on the surface of the processed materials to be joined. The microtexture covered 50% of the area intended for gluing and was distributed on a square-shaped surface with the side of 12 mm. The diameter of a single A-type texture element on the PC and aluminum surfaces was about 1 mm. However, their mean depth was 58 µm for PC and 30 µm for aluminum. Their distribution and density were selected on the basis of experimental research conducted by the authors of this article. Both A and E type textures were made on both surfaces of the combined samples. Figure 4, Figure 5 and Figure 6 show selected photographs with geometrical measurements of microstructure surfaces, taken a Hirox KH-8700 digital microscope (Hirox Co., Ltd., Tokyo, Japan) with embedded software for geometrical analysis of the examined surface.

For aluminum samples, an additional analysis of the surface condition was performed using a Joel 7100f scanning electron microscope (JEOL Ltd., Tokyo, Japan). The results are shown in Figure 7.

The research on the distribution of elements on the surface by means of the so-called “mapping” with the EDS detector did not show any noticeable changes in the distribution of elements on the surface of the tested sample, which were the result of laser treatment, which could deteriorate the adhesive properties of the tested material. Moreover, based on the SEM observation, together with the qualitative analysis of the distribution of elements on the surface of the processed material, it can be concluded that the laser modification performed did not cause any thermal damage to the surface, on the contrary, it increased the adhesion by developing it. The performed treatment increases the surface area of the material on which the glue adheres. Processing without leaving any contamination on the surface of the material is essential for the correct gluing process. In the case of thermal damage, oxides would be visible on the surface of the material, reducing the strength of the joint. The oxides remaining on the surface of the processed material are unstably bound to it. In the process of gluing, the glue, instead of permanently adhering to the material, adheres to oxides, which can easily detach from the surface of the material. In such a situation, it is often the case that such a joint is less durable than an untreated joint.

### 3.2. Material Surface Preparation

Regardless of the type of adhesive used, surface preparation of the materials to be joined is crucial to ensure a permanent and stable adhesive joint, as the strength of the joint is largely determined by the degree of adhesion between the substrate and the adhesive. To ensure an optimal joint, all kinds of dirt on the surface of the materials must be removed. Some adhesives can join through surface contamination, while others may require a thorough precleaning process prior to joining. Before the laser micromachining process could be commenced, each surface of the sample was carefully cleaned with isopropanol to remove any contamination. During the micromachining process, a surface shield in the form of a gas (as shown in Table 1) blower was used, which had two functions—it assisted the discharge of the removed material to the local suction and cooled the surface of the processed material. Additionally, in the case of aluminum, the argon used protected the material surface against excessive oxidation. After the micromachining process, the samples were cleaned with compressed air to get rid of any debris. Then, the samples were cleaned in an ultrasonic cleaner in a demineralized water solution, thus removing the ablation residues accumulated on the surface of the processed material.

The samples prepared this way were glued in a specially designed device, which ensured a constant and reproducible thickness of the obtained adhesive joint of 1 mm. Figure 8 shows the pictures of the samples during the subsequent phases of the gluing process with the use of a grip stabilizing their position in relation to each other. Figure 8A shows one part of the sample placed and stabilized in the holder, Figure 8B shows samples pressed with a spacer and application of glue, and Figure 8C shows a second part of the sample placed in the spacer and pressed onto the area of the joint. The holder enables the simultaneous gluing of five samples. The distance insert ensures the assumed thickness of the adhesive layer of 1 mm. After applying the glue, the samples were left immobilized in the holder (Figure 8) until the glue set completely (about 24 h according to the manufacturer’s declaration). The surface preparation was in accordance with the requirements of the adhesive manufacturer. In the shown connection scheme, in Figure 8D, 1 and 3 are the textured sample, 2 is the glue, and 4 is the textured area on the joined samples. The pressure is marked with arrows. Figure 9 shows a transverse view from the Al-PC joint area, where it can be seen that the adhesive penetrates the laser-made microtexture, which significantly increases the adhesive strength of the joint made. The glued samples were placed in the INSTRON 4502 machine and strength tests were performed.

### 3.3. Strength Tests

The tensile test consisted of uniaxial deformation of the samples and the measurement of forces. This method is one of the basic sources of information about the mechanical properties of plastics and adhesive joints. The value measured during the longitudinal deformation test is the breaking force of the connection. The tensile strength is therefore the maximum stress that the material will transfer on static stretching. The tests were performed on sets of five samples: with and without microstructure. 

The graphs below present the course of the tensile force of the created connection. The samples got brittle and no plastic flow of the sample was observed, both in the case of the applied binder and the tested material. The diagram of the overlapping joined elements prepared for the strength tests is shown in Figure 10, where 1 is the textured zone and 2 is the gripping part of the sample. The texture was made on the area of 12 mm × 12 mm of the joined elements and it was an overlapping glued surface. The textured surface was covered with glue and overlapped as shown in the Figure 10. Samples for static tensile tests were prepared in compliance with PN-EN ISO 6892-1 and PN-EN ISO 9664: 2000 standards.

## 4. Discussion

Table 4 shows the results of tests to determine the breaking strength of joints and the exact values of the increase in strength of the joint with the texture versus the joint without it. Variant 1 is a combination of Al and PC without texture. Variant 2 is a combination of Al with texture type A with PC with texture type E. Variant 3 is a combination of Al with texture type E with texture of PC type E. Variant 4 is a combination of Al with texture type A with PC with texture type A.

**Figure 11:**
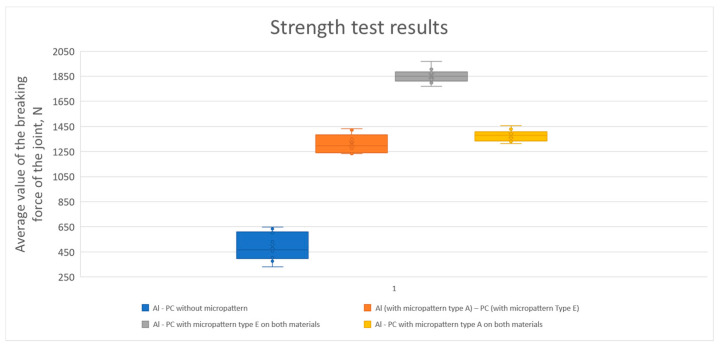
Strength test results.

**Figure 12:**
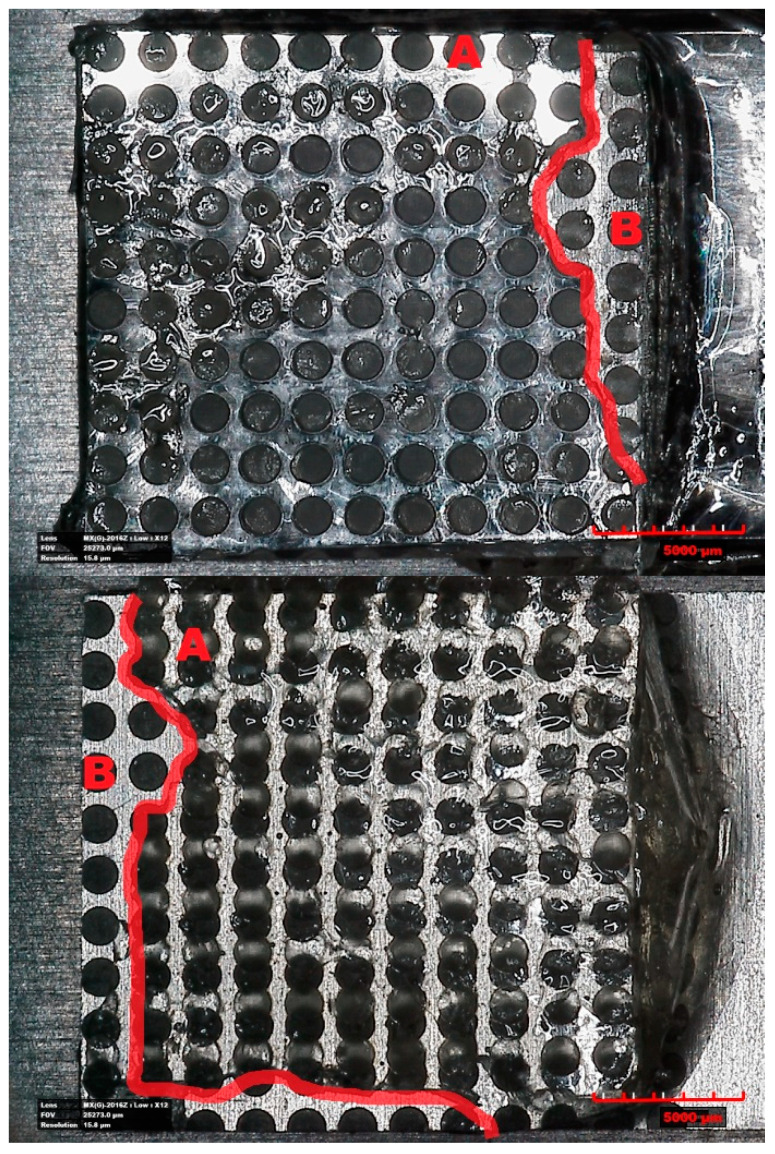
An example view of a Variant 4 sample from Table 4 after strength tests.

Figure 11 shows the results of the strength tests. Figure 12 shows an exemplary view of the microtexture made on the surface of Al and PC after tearing, together with the adhesive residues. The manufacturer of the adhesive declares that the adhesive is suitable for bonding aluminum with hard polymers such as PC.

The mechanism of breaking the connection shown in Figure 12 has an adhesive-cohesive character. A is the area of cohesive fracture and B is the area of adhesive fracture. It is the dominant nature of the breaking of the textured joint for all strength tests performed. Microscopic observations of the fracture made after the performed strength tests showed that in most cases the detachment was greater on the Al surface than on the PC. This may indicate a better adhesion of the adhesive to the polymer material, probably due to the molecular bonds of the polymer particles.

The samples joined adhesively without microtexture were broken with an average tensile force of 491.6 N. The samples adhesively joined to the circular structure on the aluminum surface and parallel lines on the PC surface were torn with an average force of 1312.1 N. The average force needed to break the sample in this variant is 266.9% greater. The samples adhesively joined to the structure in the shape of parallel lines on the surface of both joined materials were torn with an average force of 1851.7 N. The average force needed to break the adhesively joined sample in this variant is higher by 376.67%, which is the largest increase among the analyzed variants. The samples adhesively joined to the circle-shaped structure on the surface of both joined materials were torn with an average force of 1378 N. The average force needed to break the sample in this variant is greater by 280.31%.

The laser-made microtexture increases the surface area of the material [47], which has a significant impact on the force needed to break the created joint. The development of the surface increases the adhesive component of the joint made. Additionally, making a surface characterized by recesses with almost perpendicular edges in relation to the treated surface increases the mechanical component of the constituted adhesive joint. The use of such surface modification increases the force necessary to break the joint, and thus increases its strength. Unfortunately, deviations from the assumed shape resulting from material heterogeneity or surface features change the value of the contact angle, and subsequently the material surface energy.

Experimental studies preceding the results presented here have shown that a slight change in the arrangement of microtexture elements or its dimensions may cause a change in the hydrophobic or hydrophilic properties of the tested materials. The microtextures selected for this study were easy to manufacture and were executed correctly, with adequate wettability after laser modification of the surface of the tested materials. The authors are intending to perform an additional series of tests on subsequent materials, which will allow for a more detailed analysis of the obtained results. Despite the slight discrepancies obtained, these are very promising results in the perspective of further research to modify the shape, size and density of the microtexture produced.

## 5. Conclusions

The conducted research proves that the type of microtexture has a significant impact on the ability to make a more durable adhesive joint with the use of laser microtexturing on the surface of the joined material. For the tested microtexture variants, the connections obtained show a greater tear resistance during the static tensile strength test than the identical combination of materials joined without any surface structuring. The microstructures created increase the active surface area from 10% to 22%. The formation of the microstructure on the Al surface causes a significant increase in the surface free energy (SEP) [48], while for PC, depending on the type of texture, the SEP is increased or decreased [49]. The strength of a textured joint can be increased by up to 376% compared to a nontextured joint [50].

Subsequently, further studies have been scheduled to modify the shape and density of the microtexture on the surface in order to increase the strength of the adhesive joint or change the adhesive agent. More strength tests of adhesive joints for other pairs of materials are also intended: metal–plastic; ceramic–plastic and metal–ceramic, with or without a textured surface, and with or without the use of any additional adhesive.

## Figures and Tables

**Table 1:** Operating parameters of the TruMicro 5325c laser device used to make five microtexture variants.

Tested Material	Pulse Energy (µJ)	Pulse Repetition Rate (kHz)	Scanning Speed (mm/s)	Shielding Gas
Aluminum AW7075-T6 (Al)	12.6	200	250	Argon
Polycarbonate (PC)	12.6	200	1000	Air

**Table 2:** Geometrical characteristics of individual surface structures.

Texture Type	Texture Characteristics				
	Shape of a Single Texture Element	Average Dimensions of a Single Texture Element (µm/µm^2^/µm^3^)		Texture Density (%)	Degree of Surface Development (%)
Type A	Truncated cone	Depth	58	50	15.05
		Base diameter	1000		
		Volume	31,953,429		
Type B	Cuboid with a square base	Depth	53.2	50	19.15
		Side length	900		
		Volume	34,156,903		
Type C	Cuboid with the base of an equilateral triangle	Depth	54.8	50	12.09
		Side length	1360		
		Volume	25,117,254		
Type D	Inverted pyramid (square-based pyramid)	Depth	225.5	50	9.6
		Base width	900		
		Volume	68,860,529		
Type E	Crevice with an isosceles triangle cross-section	Depth	25.9	50	21.7
		Base width	53.3		
		Cross-sectional area	728		

**Table 3:** The results of the SEP measurements and the contact angle.

Tested Material	Geometric Shape of the Laser-Made Microtexture						
		Reference Sample	Type A	Type B	Type C	Type D	Type E
Aluminum AW7075-T6	Average contact angle (°)	73	32	55	50	63	20
	Average SEP (mJ/m^2^)	56.32	66.54	59.46	60.73	56.84	77.26
PC	Average contact angle (°)	87	87	89	92	83	77
	Average SEP (mJ/m^2^)	30.71	31.06	29.36	27.84	24.53	33.53

**Table 4:** Summary of the obtained strength tests results.

Strength Tests Results	Variant 1 Al—PC without Micropattern	Variant 2 Al (with Micropattern Type A)—PC (with Micropattern Type E)	Variant 3 Al—PC with Micropattern Type E on both Materials	Variant 4 Al—PC with Micropattern Type A on both Materials
Average breaking force (N)	491.6	1312.1	1851.7	1378
Dev. Std. (N)	109.18	77.97	56.55	45.42
Min (N)	332	1232	1769	1313
Max (N)	648	1432	1967	1456
The average increase in strength (%)	-	266.90	376.67	280.31

## Data Availability

Not applicable.

## References

[1] Courtney P. (2007). Joining metal with adhesives—Advantages, applications, and precautions. Fabricator.

[2] Loushin S. (2019). Structural adhesives: A viable alternative to mechanical fasteners—Open up the design possibilities with a consideration of adhesives. Fabricator.

[3] Allen K.W. (2003). “At forty cometh understanding”: A review of some basics of adhesion over the past four decades. Int. J. Adhes. Adhes..

[4] Larsson J. (2007). Laser welding, structural adhesive bonding, for body-in-white assembly. Competing or complementary joining methods?. Fabricator.

[5] Rudawska A. (2013). Wybrane Zagadnienia Konstytuowania Połączeń Adhezyjnych Jednorodnych I Hybrydowych.

[6] Zitoune R., Collombet F. (2007). Numerical prediction of the thrust force responsible of delamination during the drilling of the long-fibre composite structures. Compos. Part A Appl. Sci. Manuf..

[7] Davim J.P., Reis P., Conceição António C. (2004). Experimental study of drilling glass fiber reinforced plastics (GFRP) manufactured by hand lay-up. Compos. Sci. Technol..

[8] Matsuzaki R., Shibata M., Todoroki A. (2008). Improving performance of GFRP/aluminum single lap joints using bolted/co-cured hybrid method. Compos. Part A Appl. Sci. Manuf..

[9] Kłysz S. (2015). Charakterystyki Wybranych Materiałów—Materiały Lotnicze, 2015, Publishing House—Air Force Institute of Technology.

[10] Szlezyngier W., Brzozowski Z.K. (2012). Tworzywa Sztuczne TOM 2.

[11] Lippert T. (2004). Laser application of polymers. Adv. Polym. Sci..

[12] Chichkov B.N., Momma C., Nolte S., von Alvenslebe F., Tünnermann A. (1996). Femtosecond picosecond and nanosecond laser ablation of solids. Appl. Phys. A.

[13] Ravi-Kumar S., Lies B., Zhang X., Lyu H., Qin H. (2019). Laser ablation of polymers—A review. Polym. Int..

[14] Lippert T., Miotello A., Ossi P.M. (2010). UV laser ablation of polymers: From structuring to thin film deposition. Laser-Surface Interactions for New Materials Production.

[15] Tofil S., Antoszewski B., Mulczyk K. (2020). The efficiency of UV picosecond laser processing in the shaping of surface structures on elastomers. Polymers.

[16] Makropoulou M., Serafetinides A.A., Skordoulis C.D. (1995). Ultra-violet and infra-red laser ablation studies of biocompatible polymers. Lasers Med. Sci..

[17] Ham S.S., Lee H. (2020). Development of method enhanced laser ablation efficiency according to fine curvature of the polymer through the preliminary preparation process using UV picosecond laser. Polymers.

[18] Lasagni A.F., Roch T., Berger J., Kunze T., Lang V., Beyer E. (2015). To use or not to use (direct laser interference patterning), that is the question. Proc. SPIE.

[19] Brown M.S., Arnold C.B. (2010). Laser Precision Microfabrication.

[20] Momma C., Nolte S., Chichkov B.N., Alvensleben F.V., Tünnermann A. (1997). Precise laser ablation with ultrashort pulses. Appl. Surf. Sci..

[21] Antoszewski B. (2010). Textured Surface Layers-Shaping with Selected Beam Technologies and Tribological Properties.

[22] Radek N., Bartkowiak K. (2012). Laser treatment of electro-spark coatings deposited in the carbon steel substrate with using nanostructured WC-Cu electrodes. Phys. Procedia.

[23] Madej M. (2014). The effect of TiN and CrN interlayers on the tribological behavior of DLC coatings. Wear.

[24] Gądek-Moszczak A., Radek N., Wroński S., Tarasiuk J. (2014). Application the 3D image analysis techniques for assessment the quality of material surface layer before and after laser treatment. Adv. Mater. Res..

[25] Witkowski G., Tofil S.Z., Mulczyk K. (2020). Effect of laser beam trajectory on pocket geometry in laser micromachining. Open Eng..

[26] Alamri S., El-Khoury M., Aguilar-Morales A.I., Storm S., Kunze T., Lasagni A.F. (2019). Fabrication of inclined non-symmetrical periodic micro-structures using direct laser interference patterning. Sci. Rep..

[27] Fraggelakis F., Mincuzzi G., Lopez J., Manek-Hönninger I., Kling R. (2018). Controlling 2D laser nano structuring over large area with double femtosecond pulses. Appl. Surf. Sci..

[28] Mezera M., van Drongelen M., Römer G.R.B.E. (2018). Laser-induced periodic surface structures (LIPSS) on polymers processed with picosecond laser pulses. J. Laser Micro/Nanoeng..

[29] Romano J.M., Garcia-Giron A., Penchev P., Dimov S. (2018). Triangular laser-induced submicron textures for functionalising stainless steel surfaces. Appl. Surf. Sci..

[30] Zhai T., Zhang X., Pang Z., Dou F. (2011). Direct writing of polymer lasers using interference ablation. Adv. Mater..

[31] Bityurin N., Luk’yanchuk B.S., Hong M.H., Chong T.C. (2003). Models for laser ablation of polymers. Chem. Rev..

[32] Bityurin N. (2005). Studies on laser ablation of polymers. Annu. Rep. Prog. Chem..

[33] Garcia-Giron A., Romano J.M., Batal A., Dashtbozorg B., Dong H., Solanas E.M., Dimov S.S. (2019). Durability and wear resistance of laser-textured hardened stainless steel surfaces with hydrophobic properties. Langmuir.

[34] Lippert T. (2005). Interaction of photons with polymers: From surface modification to ablation. Plasma Process. Polym..

[35] Antoszewski B., Tofil S., Scendo M., Tarelnik W. (2017). Utilization of the UV laser with picosecond pulses for the formation of surface microstructures on elastomeric plastics. Iop Conf. Ser. Mater. Sci. Eng..

[36] Serafetinides A.A., Makropoulou M.I., Skordoulis C.D., Kar A.K. (2001). Ultra-short pulsed laser ablation of polymers. Appl. Surf. Sci..

[37] Zhai T., Wang Y., Liu H., Zhang X. (2015). Large-scale fabrication of flexible metallic nanostructure pairs using interference ablation. Opt. Express.

[38] Liu H.B., Gong H.Q. (2009). Templateless prototyping of polydimethylsiloxane microfluidic structures using a pulsed CO_2_ laser. J. Micromech. Microeng..

[39] Liu Z.Q., Feng Y., Yi X.S. (2000). Coupling effects of the number of pulses, pulse repetition rate and fluence during laser PMMA ablation. Appl. Surf. Sci..

[40] Mao B., Siddaiah A., Liao Y., Menezes P.L. (2020). Laser surface texturing and related techniques for enhancing tribological performance of engineering materials: A review. J. Manuf. Process..

[41] Romano J.M., Gulcur M., Garcia-Giron A., Martinez-Solanas E., Whiteside B.R., Dimov S.S. (2019). Mechanical durability of hydrophobic surfaces fabricated by injection moulding of laser-induced textures. Appl. Surf. Sci..

[42] Zheng H.Y., Guan Y.C., Liu K., Wang Z.K., Yuan S.M. (2016). Other methods of polymer surface modifications printing on polymers. Fundam. Appl..

[43] Lugscheider E., Bobzin K. (2001). The influence on surface free energy of PVD-coatings. Surf. Coat. Technol..

[44] Vedantam S., Panchagnula M.V. (2008). Constitutive modeling of contact angle hysteresis. J. Colloid Interface Sci..

[45] Zielecka M. (2004). Methods of contact angle measurement as a tool for characterization of wettability of polymers. Polimery.

[46] Rudawska A., Jacniacka E. (2009). Analysis of determining surface free energy uncertainty with the owens-wendt method. Int. J. Adhes. Adhes..

[47] Liu X.B. Industrial applications of ultrahigh precision short-pulse laser processing. Proceedings of the Microelectronics and Photonics IV.

[48] Wu B., Zhou M., Li J., Ye X., Li G., Cai L. (2009). Superhydrophobic surfaces fabricated by microstructuring of stainless steel using a femtosecond laser. Appl. Surf. Sci..

[49] Cardoso M.R., Martins R.J., Dev A., Voss T., Mendonca C.R. (2015). Highly hydrophobic hierarchical nanomicro roughness polymer surface created by stamping and laser micromachining. J. Appl. Polym. Sci..

[50] Sawa T., Liu J., Nakano K., Tanaka J. (2000). A two-dimensional stress analysis of single-lap adhesive joints of dissimilar adherends subjected to tensile loads. J. Adhes. Sci. Technol..

